# Genetics of Myasthenia Gravis: A Case-Control Association Study in the Hellenic Population

**DOI:** 10.1155/2012/484919

**Published:** 2012-09-25

**Authors:** Zoi Zagoriti, Marianthi Georgitsi, Olga Giannakopoulou, Fotios Ntellos, Socrates J. Tzartos, George P. Patrinos, Konstantinos Poulas

**Affiliations:** ^1^Laboratory of Molecular Biology and Immunology, Department of Pharmacy, School of Health Sciences, University of Patras, Rion, 26504 Patras, Greece; ^2^Department of Biochemistry, Hellenic Pasteur Institute, 127 Vassilissis Sofias Avenue, 11521 Athens, Greece

## Abstract

Myasthenia gravis (MG) is an heterogeneous autoimmune disease characterized by the production of autoantibodies against proteins of the postsynaptic membrane, in the neuromuscular junction. The contribution of genetic factors to MG susceptibility has been evaluated through family and twin studies however, the precise genetic background of the disease remains elusive. We conducted a case-control association study in 101 unrelated MG patients of Hellenic origin and 101 healthy volunteers in order to assess the involvement of common genetic variants in susceptibility to MG. We focused on three candidate genes which have been clearly associated with several autoimmune diseases, aiming to investigate their potential implication in MG pathogenesis. These are interferon regulatory factor 5 (IRF-5), TNF**α**-induced protein 3 (TNFAIP3), also known as A20, and interleukin-10 (IL-10), key molecules in the regulation of immune function. A statistical trend of association (*P* = 0.068) between *IL-10* promoter single nucleotide polymorphisms (SNPs) and the subgroups of early and late-onset MG patients was revealed. No statistically significant differences were observed in the rest of the variants examined. As far as we are aware, this is the first worldwide attempt to address the possible association between *IRF-5* and *TNFAIP3* common genetic variants and the genetic basis of MG.

## 1. Introduction

Myasthenia gravis (MG) is an organ-specific autoimmune disease caused by autoantibodies directed against proteins of the postsynaptic membrane leading to impaired neuromuscular transmission. Clinically, MG is characterized by muscle weakness and rapid fatigue aggravated by exercise and relieved by rest. The main autoantigen, in 80–90% of MG patients, is the muscle acetylcholine receptor (AChR), a pentameric channel which mediates synaptic transduction at the neuromuscular junction [1]. In several of the remaining MG patients, autoantibodies to muscle-specific tyrosine kinase (MuSK) [2] or to lipoprotein-related protein 4 (LRP4) [3] are detected. Both MuSK and LRP4 form a receptor complex which binds the extracellular matrix proteoglycan agrin, resulting in AChR clustering, critical for neuromuscular junction function.

Although MG is a disease that affects both sexes, at all ages and in all races [4], evidence from several epidemiological studies have showed a sex- and age-dependent bimodal distribution of incidence rate, with one peak in the second and third decades of life, observed mostly in women, and the second one in the sixth and seventh decades of life occurring mainly in men [5]. The above observation led to the classification of MG into early onset which appears before 50 years of age and is usually related to thymus hyperplasia and late-onset MG (>50 years) with normal or atrophic thymus.

The extent of genetic contribution to MG susceptibility has been evaluated through family [6–8] and twin studies [9], reflective of the disease's familial clustering and subsequently of genetic inheritance. High concordance rates of MG observed among monozygotic twins compared with dizygotic twins strongly suggest the involvement of genetic factors in the pathogenesis of MG [9]. Moreover, several studies have reported that MG patients may be affected with another autoimmune disease, most frequently, thyroid disorders and rheumatoid arthritis [10]. This finding leads to the hypothesis that a more generalized disturbance of the immunological function occurs. 

The human leukocyte antigen (HLA) complex is the prominent genomic region implicated in MG onset. HLA-A1 and B8 alleles for class I and DR3 for class II constitute an ancestral haplotype termed “8.1” which has been reproducibly associated with early onset MG and thymic hyperplasia [11]. Further research geared towards the dissection of this extended A1-B8-DR3 haplotype, led to the identification of the MYAS1 locus, a region of 1.2 Mb encompassing 36 genes, at the boundary of class III and proximal class I region, thus, excluding the class II loci and confirming the predominant association of B8 allele over that of DR3 [12]. 

Apart from the HLA, a number of HLA-unlinked genetic loci have also been investigated regarding their involvement in MG susceptibility. These findings have mainly been derived by candidate gene studies, while the genes that were reported to be associated or not associated with MG are discussed in detail in [13]. 

Interferon (IFN) regulatory factor 5 (IRF-5) is a member of the IRF family of transcription factors. IRF-5 is activated by IFN-*α*/B and upregulates a set of proinflammatory cytokines, such as IL-6, TNF-*α*, and IL-12, while it further induces IFN gene expression. Results from several studies, reviewed in [14], have implicated *IRF-5* as a susceptibility gene in SLE. Search for common variants that influence IRF-5 levels led to the identification of SNP rs10954213 (c.*555G > A), located within the polyA+ signal sequence AATAAA in the 3′ UTR. The G allele disrupts the polyadenylation site and transcription is terminated far downstream, thus producing longer and less stable IRF-5 mRNA transcripts [15]. Further, a 30-bp in-frame insertion-deletion variant (rs60344245) in the sixth exon of *IRF-5* determines the formation of two families of protein isoforms which have differential ability to initiate transcription of IRF-5 target genes [16]. 

The TNF*α*-induced protein 3 (TNFAIP3), also known as A20, is a key molecule in the negative feedback regulation of NF-*κ*B-dependent responses. The inhibitory effect of TNFAIP3 on NF-*κ*B signaling is generated from the cooperative activity of its two ubiquitin-editing domains: the N-terminal ovarian tumor domain (OTU), responsible for deubiquitinating receptor interacting protein 1 (RIP1), an essential adaptor protein of the TNF-induced signaling pathway, and the C-terminal zinc finger-containing domain, which functions as an E3 ubiquitin ligase promoting the proteasomal degradation of RIP1 [17]. 

The SNP rs13207033 (g.137965418G > A), located at 6q23 intergenic region, approximately 185 kb upstream of *TNFAIP3*, probably affects gene expression by the presence of potential regulatory DNA elements [18]. Another study indicated that a nonsynonymous coding SNP (c.380T > G, rs2230926) resulting in a phenylalanine-to-cysteine change at residue 127 (p.F127C), in the OTU domain of the TNFAIP3 protein, is associated with SLE among individuals of European ancestry [19]. 

Interleukin-10 (IL-10) is a pleiotropic cytokine secreted by different cell types, such as T cells and myeloid lineage cells. IL-10 has been characterized as an anti-inflammatory cytokine due to its stimulatory effects on T_H_2 cells [20] and to the simultaneous suppression of T_H_1 cells [21]. Moreover, IL-10 induces proliferation and differentiation of activated B lymphocytes [21] leading to further activation of humoral response. In experimental autoimmune MG (EAMG), IL-10 administration caused the increase of anti-AChR antibody levels, suggesting a disease-enhancing role of IL-10 [22]. 

Several variants have been noticed in the 5′ flanking sequence of the human *IL-10* gene. Three SNPs, namely, rs45552637 (A/C), rs1800872 (T/C), and rs1800896 (A/G), located at positions −592, −819, and −1082, respectively, determine the formation of three haplotypes (GCC, ACC, and ATA). The position of these SNPs is based on the previously published sequence U16720, deposited in the EMBL-EBI database. A study by Turner and coworkers reported correlation of these haplotypes with IL-10 protein production *in vitro *[23]. Specifically, GCC/GCC genotype was associated with high concanavalin A-induced IL-10 production, GCC/ACC and GCC/ATA genotypes with medium and ACC/ACC, ATA/ATA, and ACC/ATA genotypes with low IL-10 production. 

In the current study, a hypothesis-driven approach was adopted in order to assess the involvement of certain common variants in MG susceptibility. Thus, we conducted a candidate gene case-control study, focusing on genes with a critical role in immune system function, aiming to identify whether previously reported associations between the above genes and other autoimmune diseases could hold true for MG. 

## 2. Materials and Methods

### 2.1. Study Population

A total of 101 unrelated MG patients, all of Hellenic descent, were enrolled in this study. Blood samples from MG patients were collected at the Hellenic Pasteur Institute during routine diagnostic survey. Only AChR-positive MG patients were included in our study. The diagnosis of MG was based on the presence of anti-AChR antibodies in the patient's serum, using a radioimmunoprecipitation assay (RIPA). We intentionally excluded from the genetic analysis those patients who were identified as positive for autoantibodies against MuSK, in order to reduce the heterogeneity of the study group. Although sera of MG patients were not analyzed for anti-LRP4 autoantibodies, the absence of this test raises no issue of heterogeneity in the study population, because of the rare coexistence of anti-LRP4 and anti-AChR antibodies. The main characteristics of the anti-AChR MG group (age at onset and gender) are summarized in Table 1. Written informed consent was obtained by patients. The control group consisted of 101 ethnically and sex-matched healthy individuals. 

### 2.2. Genotyping

Genomic DNA from each individual was extracted from peripheral venous blood sample using the QIAamp Blood Midi kit (Qiagen GmbH, Hilden, Germany). Polymerase chain reactions (PCRs) were performed with the KAPA2G Fast HotStart ReadyMix kit (KAPABIOSYSTEMS, Woburn, MA, USA). Primer sequences are presented in the supplementary materials as Supplementary Table 1 (see Table 1 in Supplementary Material available online at doi:10.1155/2012/484919), whereas reaction conditions are available upon request.

Amplification by PCR and agarose gel electrophoresis analysis were used to genotype the 30 bp insertion/deletion variant of *IRF5.*

PCR-based restriction fragment length polymorphism (RFLP) assay was used for the detection of SNP rs2230926 (T > G) in *TNFAIP3*. The amplified fragments of 549 bp were digested with the restriction enzyme *Fnu4HI *(New England Biolabs, Ipswich, MA, USA) and were then analyzed by electrophoretic separation on 2% w/v agarose gel. The G allele creates an *Fnu4HI* restriction site, resulting in the digestion of amplicons to 319 and 230 bp fragments. 

 The identification of *IL-10 *promoter SNPs genotypes was performed by direct DNA Sanger sequencing. A fragment of 585 bp, including all three variants, was amplified by PCR. The PCR products were purified by the column-based PureLink PCR Purification kit (Invitrogen, Carlsbad, CA, USA). The sequence of the 585 bp fragment was determined using the BigDye Terminator chemistry v3.0 kit on an Applied Biosystems 3730 × l DNA sequencer (Applied Biosystems, Carlsbad, CA, USA). The primers used were the same as those for the amplification of this region. 

Both SNPs, rs10954213 (A/G) in the 3′ UTR of *IRF5 *and rs13207033 (G/A) located at a 6q23 intergenic region, were genotyped by real-time PCR and high resolution melting curve (HRM) analysis on a RotorGene Q real-time cycler (Qiagen GmbH, Hilden, Germany). The amplification of the fragment containing the SNP of interest was carried out using the Type-it HRM PCR kit (Qiagen GmbH, Hilden, Germany), according to manufacturer instructions. During HRM, the temperature increases from 65 to 95°C, with a heating rate of 0.1°C/2 sec, leading to the denaturation of PCR products and the generation of melting curves, characteristic for each genotype. Since a single base-pair change causes a significant shift in the melting temperature (*T*_*m*_), genotyping is based on the analysis of the melting profiles: homozygotes for the A allele exhibit similar melting profiles and with a lower *T*_*m*_, compared with the G/G homozygotes, whereas heterozygotes are differentiated by a change in the shape of the melting curve.

No template controls were meticulously included in all genotyping processes. Negative and positive control samples were initially identified by DNA sequencing and were, subsequently, used in all genotyping methods. Each sample was tested in duplicate, except for those analyzed by PCR-RFLP and DNA sequencing.

### 2.3. Statistical Analysis

The differences in genotype distribution and allele frequencies between cases and controls were calculated by *χ* ^2^ analysis or Fisher's Exact test. *P* values less than 0.05 were regarded as statistically significant.

## 3. Results

Genotype distributions of all variants were consistent with Hardy-Weinberg equilibrium in both MG patient and control groups (data not shown). 

The allele and genotype frequencies of the *IRF-5 *rs60344245 variant showed an akin distribution in the screened groups of 101 MG patients and 100 controls (*P* = 0.76). Regarding the *IRF-5* rs10954213 SNP, A/G genotype was found to be somewhat more frequent in MG patients than in controls (54.8% versus 45.5%), but *χ* ^2^ analysis revealed no significant difference (*P* = 0.3). Allele and genotype frequencies of both *IRF-5 *variants are shown in Table 2. 

The *TNFAIP3* rs13207033 G/G genotype frequency showed an increase in healthy controls (44.6%) compared to MG patients (33.3%). However, statistical analysis did not indicate any significant difference between the two groups (*P* = 0.17). In the case of rs2230926 coding SNP, genotypes are distributed similarly in the 73 MG patients and 81 controls examined, as it is inferred by the *P* value = 0.74. Genotype frequencies of the rs2230926 variant, in both MG patients and controls, are in accordance with those derived from samples of European ancestry (CEU) that are part of the international HapMap project. In addition, our study group exhibited rs13207033-genotype frequencies which are comparable to the frequencies reported in European populations, in the dbSNP database. Genotyping results of the two *TNFAIP3 *variants are summarized in Table 3. 

Age of disease onset was also evaluated by dividing MG patients into early- and late-onset patients. No significant difference in genotype distribution was detected between the two subgroups and the control group (data not shown). 

**Figure 1:**
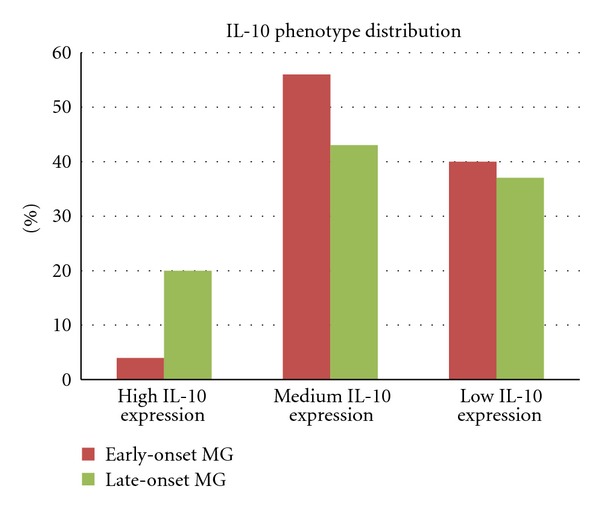
*IL-10 *phenotype distribution in the subgroups of early- and late-onset MG patients.

DNA sequence analysis of *IL-10* promoter region showed that the ACC/GCC genotype was the most frequently observed genotype in both MG patients and controls (23.72% and 28%, resp.), followed by the low secretion genotype ATA/ACC, which was detected in 21.62% of total MG and 18% of controls (Table 4). However, the current study did not reveal any statistically significant difference in *IL-10* genotype distribution between the complete cohort of MG patients (i.e., total MG) and the control group (*P* = 0.7). Comparison between the subsets according to age at onset demonstrated that the high IL-10 secretion GCC/GCC genotype is found in a low frequency in early-onset MG (4%), while it is overrepresented in late onset MG cases (20%) (Table 4). A statistical trend of association (*P* = 0.068) between the IL-10 phenotype distribution and the two subgroups of early and late onset was revealed (Figure 1).

## 4. Discussion 

MG is a heterogeneous autoimmune disease with a clear genetic predisposition. In addition to the HLA loci, several common variants in HLA-unlinked genes have been associated with MG susceptibility [13]. Many of these risk-associated genes are widely distributed among various autoimmune diseases, supporting the notion that autoimmune diseases are characterized by shared pathogenetic pathways. In this study, we performed a case-control association study in order to investigate the contribution of common variants located in *IRF-5*, *TNFAIP3*, and *IL-10* genes to MG susceptibility. These genes were considered as good candidates because of their critical role in the regulation of immune response and their previously known implication in the autoimmune process [24]. Only patients with anti-AChR antibodies in their serum were included in the genetic analysis, as it has been expected that they represent a more homogenous subset than the broader MG group. This subgroup was further divided into two distinct disease entities: early-onset MG patients, comprising mostly women, and late-onset MG patients, showing a higher proportion of men.

As far as we are aware, this is the first study, in any population, to investigate the association between MG and common variants of *IRF-5* and *TNFAIP3* genes. According to previous studies, reviewed in [14], several variants in the *IRF-5* locus have been reproducibly associated with SLE implicating *IRF-5* as a susceptibility gene in lupus. The rs10954213 SNP has been found to influence mRNA polyadenylation, thus, impairing the levels of IRF-5 protein; A/A homozygotes express approximately 5-fold higher levels of immunoreactive IRF-5 compared to the G/G homozygotes [25]. As for the rs60344245 variant, the deletion of 30 bp (GGCCGCCTACTCTGCAGCCGCCCACTCTGC/−) removes 10 amino acids from the IRF-5 protein and alters a proline-, glutamic acid-, serine-, and threonine-rich (PEST) domain. In the IRF family of proteins, such domains participate in protein interactions [26] and they also cause rapid proteolytic degradation [27]. Despite their obvious functional role, the current study failed to demonstrate a significant association of the *IRF-5 *rs10954213 and rs60344245 variants with MG (*P* = 0.3 and *P* = 0.76, resp.), suggesting that *IRF-5* may not be involved in MG pathogenesis.

Furthermore, recent findings from GWA studies have revealed significant associations between variants in the human *TNFAIP3* locus and a wide spectrum of autoimmune diseases. A GWA scan of rheumatoid arthritis patients, with anticitrullinated peptide antibodies, detected strong evidence of association of the rs13207033 SNP with the development of RA [28]. Similarly, a study by Musone and coworkers reported the association of the nonsynonymous coding SNP, rs2230926 with SLE [19]. Functional studies to determine the biological impact of rs2230926 demonstrated that the minor Cys127 protein shows a decreased inhibitory activity [19]. Yet, lack of association was observed between MG and *TNFAIP3 *rs13207033 (*P* = 0.17) and rs2230926 (*P* = 0.74) SNPs.

Altogether, our data may indicate that the organ-specific MG might have a different genetic background leading to its separation from a wide cluster of systemic autoimmune diseases comprising SLE and RA [29]. An alternative explanation for the lack of association in our study could be related to the insufficient statistical power owing to relatively small sample sizes. As it is generally known, common variants account for a modest proportion of the genetic risk regarding the autoimmune diseases. In such cases, thousands of samples may be required in order to detect an association signal that can be distinguished from the background noise [30, 31]. However, in low prevalence diseases, such as MG, the recruitment of large sample sizes is very difficult. It is worth mentioning that the diagnostic unit of MG in the Hellenic Pasteur Institute is the only unit in Greece which has systematically received and analyzed blood samples since 1983. Therefore, our collection of MG DNA samples is currently the largest in Greece and it is constantly enriched by new cases. 

Moreover, despite the specific selection of anti-AChR patients and their division into early and late onset, a further classification according to the thymus anomalies (thymoma or hyperplasia) could have been informative; however, histological data were not available. 

Finally, an unexpected result was the lack of association of the *IL-10 *promoter SNPs with MG. Since these variants are presumed to lie within the IL-10 promoter region, they may affect the binding of transcription factors that regulate IL-10 expression. A recent study by Alseth and coworkers, conducted on the Norwegian population, revealed an association of ACC/ACC genotype with the subgroup of titin antibodies-positive MG patients, while a statistically significant increased ATA/ATA frequency was observed in early-onset MG patients [32]. In our group of Hellenic MG cases, no evidence of association was detected, when we compared the genotype distribution between the complete cohort of MG patients and the control group (*P* = 0.7). However, GCC/GCC genotype revealed a statistical trend of association with MG, in the distinct subgroups of early and late-onset MG patients (*P* = 0.068). Thus, further studies in larger sample sizes could uncover possible associations of MG with IL-10. It is also noteworthy the fact that allele frequencies for a given SNP may vary substantially across ethnic groups. The difference noticed in the frequency of the ACC/ACC genotype between the Norwegian and Hellenic control groups (3.4% versus 15%) is reflective of this condition. 

Overall, this has been the first effort, to our knowledge, to address the possible association between common genetic variants of *IRF-5 *and *TNFAIP3* and the genetic basis of MG, in any population, whereas further studies are needed to unravel the, yet largely unknown, genetic background of MG. 

## Supplementary Material

The Supplementary Material includes Supplementary Table 1 that provides information about the primers for PCR amplification of each variant and its flanking sequence.

## Figures and Tables

**Table 1:** Characteristics of the MG study group.

	Anti-AChR MG patients (*N* = 101)		
	Early onset (*n* = 45)	Late onset (*n* = 44)	Unknown age at onset (*n* = 12)
Gender (male/female)	8/37	27/17	6/6
Age at onset (mean ± SD)	32.8 ± 8.9	65.8 ± 8.6	—

SD: standard deviation.

**Table 2:** Genotype and allele distribution of IRF-5 variants in MG patients and controls. Statistical values calculated by *χ* ^2^ test are also shown.

IRF-5 variants	Control	MG	*P*_value_
rs60344245	*N* = 100 (%)*	*N* = 101 (%)	

Genotypes			
insertion/insertion	28 (28.0)	24 (23.8)	
insertion/deletion	48 (48.0)	53 (52.4)	0.76
deletion/deletion	24 (24.0)	24 (23.8)	
Alleles			
insertion	104 (52.0)	101 (50.0)	0.76
deletion	96 (48.0)	101 (50.0)	

rs10954213	*N* = 101 (%)	*N* = 84 (%)*	

Genotypes			
A/A	37 (36.7)	29 (34.5)	
A/G	46 (45.5)	46 (54.8)	0.3
G/G	18 (17.8)	9 (10.7)	
Alleles			
A	120 (59.0)	104 (62.0)	0.7
G	82 (41.0)	64 (38.0)	

*Analysis was not successful for a subset of samples.

**Table 3:** TNFAIP3 genotype and allele frequencies in MG patients and controls. Statistical values calculated by *χ* ^2^ test are also shown.

TNFAIP3 variants	Control	MG	P_value_
rs13207033	*N* = 101 (%)	*N* = 93 (%)*	

Genotypes			
G/G	45 (44.6)	31 (33.3)	
A/G	44 (43.5)	53 (57.0)	0.17
A/A	12 (11.9)	9 (9.7)	
Alleles			
G	134 (66.0)	115 (62.0)	0.41
A	68 (34.0)	71 (38.0)	

rs2230926	*N* = 81 (%)*	*N* = 73 (%)*	

Genotypes			
T/T	77 (95.1)	68 (93.2)	
T/G	4 (4.9)	5 (6.8)	0.74
G/G	0 (0.0)	0 (0.0)	
Alleles			
T	158 (98.0)	141 (97.0)	0.74
G	4 (2.0)	5 (3.0)	

*Analysis was not successful for a subset of samples.

**Table 4:** IL-10 genotype frequencies in the complete cohort of MG patients (i.e., total MG), the subgroups of early and late onset MG and controls.

Phenotype	Genotypes	Total MG *N* = 97 (%)^a^	Early onset *N* = 45 (%)^b^	Late onset *N* = 44 (%)^b^	Control *N* = 100 (%)^a^
High IL-10 expression	GCC/GCC	11 (11.32)	2 (4.0)	9 (20.0)	13 (13.0)

Medium IL-10 expression	ACC/GCC	23 (23.72)	12 (27.0)	10 (23.0)	28 (28.0)
	ATA/GCC	24 (24.72)	13 (29.0)	9 (20.0)	17 (17.0)

Low IL-10 expression	ACC/ACC	12 (12.42)	5 (11.0)	6 (14.0)	15 (15.0)
	ATA/ATA	6 (6.2)	4 (9.0)	2 (5.0)	9 (9.0)
	ATA/ACC	21 (21.62)	9 (20.0)	8 (18.0)	18 (18.0)

^a^Analysis was not successful for a subset of samples.

^b^Eight MG samples were of unknown age at onset.
