# Supplementary material for: Genetics of Myasthenia Gravis: A Case-Control Association Study in the Hellenic Population
Source: Clin Dev Immunol. 2012 Sep 25;2012:484919. doi: 10.1155/2012/484919 (PMC3463197; doi:10.1155/2012/484919)
Supplement: Supplementary file 1 — The Supplementary Material includes Supplementary Table 1 that provides information about the primers for PCR amplification of each variant and its flanking sequence. [file 484919.f1.docx]

**SUPPLEMENTARY MATERIAL**

**Supplementary Table 1:** Primers used for PCR amplification of each variant’s flanking region.

| **GENE** | **VARIANT** | **PRIMER SEQUENCE (5’ 🡪 3’)** | **PRODUCT SIZE (bp)** |
| --- | --- | --- | --- |
| ***IL-10*** | rs1800896 (-1082)  rs1800872 (-819)  rs45552637 (-592) | F: ATCCAAGACAACACTACTAA | **585** |
|  |  | R: ATCCTCAAAGTTCCCAAGCA |  |
| ***IRF5*** | rs10954213 | F: CCAAGAACCTGGAGCAGAAA | **106** |
|  |  | R: GCATGGGAGCAACTGGTAGT |  |
|  | rs60344245 | F: CCCCACATGACACCCTATTC | **180/210** |
|  |  | R: GCTCCAGGACCTCAGAGAGA |  |
| ***TNFAIP3*** | rs2230926 | F: CTCCTTTGCAGTTGGTGTCA | **549** |
|  |  | R: GCTTCGCTTAGCCAAATTCA |  |
|  | rs13207033 | F: ATTTTATGCTCCATGGGAAA | **112** |
|  |  | R: GCACAATGAAAGAGAGAGAAGTAGA |  |
